# Suppression of adiponectin receptor 1 promotes memory dysfunction and Alzheimer’s disease-like pathologies

**DOI:** 10.1038/s41598-017-12632-9

**Published:** 2017-09-29

**Authors:** Min Woo Kim, Noman bin Abid, Myeong Hoon Jo, Min Gi Jo, Gwang Ho Yoon, Myeong Ok Kim

**Affiliations:** Division of Life Science and Applied Life Science (BK 21 plus), College of Natural Sciences, Gyeongsang National University, Jinju, Republic of Korea

## Abstract

Recent studies on neurodegeneration have focused on dysfunction of CNS energy metabolism as well as proteinopathies. Adiponectin (ADPN), an adipocyte-derived hormone, plays a major role in the regulation of insulin sensitivity and glucose homeostasis in peripheral organs via adiponectin receptors. In spite of accumulating evidence that adiponectin has neuroprotective properties, the underlying role of adiponectin receptors has not been illuminated. Here, using gene therapy-mediated suppression with shRNA, we found that adiponectin receptor 1 (AdipoR1) suppression induces neurodegeneration as well as metabolic dysfunction. AdipoR1 knockdown mice exhibited increased body weight and abnormal plasma chemistry and also showed spatial learning and memory impairment in behavioural studies. Moreover, AdipoR1 suppression resulted in neurodegenerative phenotypes, diminished expression of the neuronal marker NeuN, and increased expression and activity of caspase 3. Furthermore, AD-like pathologies including insulin signalling dysfunction, abnormal protein aggregation and neuroinflammatory responses were highly exhibited in AdipoR1 knockdown groups, consistent with brain pathologies in ADPN knockout mice. Together, these results suggest that ADPN-AdipoR1 signalling has the potential to alleviate neurodegenerative diseases such as Alzheimer’s diseases.

## Introduction

Neurodegeneration is a term describing a pathological phenotype observed in the central nervous system, especially the brain^1^. Many etiological models of neurodegeneration, such as that in Alzheimer’s disease (AD) and Parkinson’s disease (PD), are based on abnormal protein aggregation and sequentially entail chronic inflammation, generation of reactive oxygen species (ROS) and apoptosis^2–4^. However, most cases of neurodegeneration are sporadic and do not result from familiar or genetic causes^5^. Therefore, neurodegenerative diseases must be identified from various research perspectives. In recent years, attempts to investigate neurodegeneration as a metabolic dysfunction, such as insulin resistance and glucose intolerance, have been made, and the efforts have gradually been recognized^6–9^. In AD clinical studies, patients with AD exhibited abnormal metabolic parameters such as hyperglycaemia and hyperinsulinaemia^10,11^. Additionally, insulin signalling, a representative pathway of energy metabolism, is highly down-regulated in the brain as well as in the periphery; therefore, these patients experience decreased glucose utilization and insulin sensitivity^12^. Hence, ameliorating energy metabolism that has been decreased by an ageing-associated disorder may have potential as a therapy for neurodegeneration before neurodegenerative proteinopathies have occurred. Indeed, several groups have tested the use of thiazolidinediones (TZDs), which are agonists of proliferator-activated gamma-type (PPAR-γ) receptors, in AD mouse models, and have observed decreased spatial learning and memory impairment and increased synaptic plasticity^13,14^. Therefore, neurodegenerative diseases have been termed type 3 diabetes (T3D), and the identification of the molecular mechanisms of ‘brain-metabolic disorder’ have gained attention in research^15^.

Adiponectin (ADPN), a fat-derived hormone, is an important player in several metabolic pathways. ADPN is involved in whole-body energy metabolism by sensing insulin in various organs^16^. Major branches of the ADPN signalling pathway activate AMP-activated protein kinase (AMPK) via adiponectin receptors (AdipoRs). This ADPN-AdipoRs-AMPK axis facilitates sequential downstream energy dissipation, lipid metabolism such as beta-oxidation and anti-atherosclerotic actions. The axis is not limited to peripheral organs. Although ADPN is derived solely from adipocytes, AdipoRs are widely distributed throughout the body. There is a difference in the expression of the AdipoRs; AdipoR1 is ubiquitously expressed, whereas expression of AdipoR2, which is 66.7% homologous to AdipoR1, is expressed mostly in the liver^17,18^. As mentioned above, efforts have been made to introduce peripheral factors into the neurodegenerative pathology. ADPN is the one of the strongest candidates to mitigate neurodegenerative diseases as well as to serve as a therapeutic target. AD patients exhibit low serum concentrations of ADPN, the rs266729/rs1501299 ANP gene polymorphisms, and the GT and CG haplotypes^19^. Additionally, ADPN ameliorates toxin-induced neuronal apoptosis such as that induced by reactive oxygen species and kainic acid in rodents^20,21^. In contrast, the basal level of neuroinflammation and apoptosis are highly up-regulated in ADPN knockout (ADPN^−/−^) mice^22^. Thus, ADPN may be a central factor in not only energy metabolism but also neuropathology.

AdipoR1 knockout (AdipoR1^−/−^) mice have been reported to exhibit higher adiposity, insulin resistance and abnormal plasma chemistry^23^. In contrast, AdipoR2^−/−^ mice show resistance to high-fat diet (HFD)-induced adiposity and insulin resistance. These opposing effects make approaching the ADPN-mediated amelioration of neurodegenerative diseases difficult. To understand the mechanisms of ADPN or ADPN-like ligands in the CNS, the physiological role of AdipoRs in the CNS must be considered. Here, we first focused on the specific role of AdipoR1 in the CNS, because ADPN exhibits selective binding to AdipoR2 but not AdipoR1^24^.

Here, we found that shRNA-mediated AdipoR1 suppression resulted in dysfunction of basal metabolism under feeding of a normal chow diet. Next, AdipoR1 knockdown (AKD) mice exhibited AD-like pathologies such as spatial and learning memory impairment and neuronal apoptosis, compared with that observed in WT mice and mouse hippocampal HT22 cells transfected with scrambled (Scr) shRNA. Our findings suggest that AdipoR1 is an essential receptor for protecting against neuronal cell death as well as spatial and learning memory impairment.

## Results

### Confirmation of AdipoR1 shRNA efficiency in an *in vivo* and ***in vitro*** model

**Figure 1:**
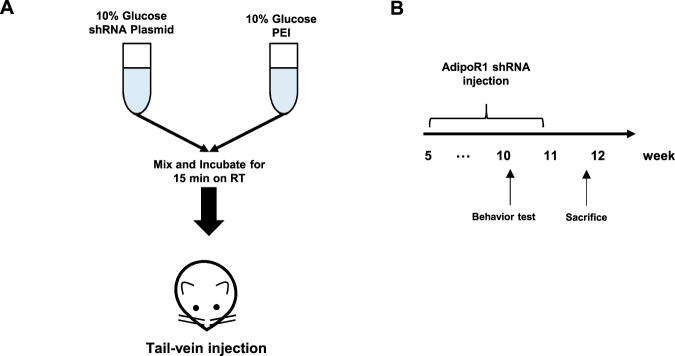
Steps involved in the preparation and injection of the *in vivo* PEI-shRNA plasmid complex into the animal model through the tail vein. (**A**) PEI and shRNA plasmid were dissolved in a 10% glucose solution and injected once weekly for 5 weeks. (**B**) Schematic diagram of PEI-shRNA experiment.

**Figure 2:**
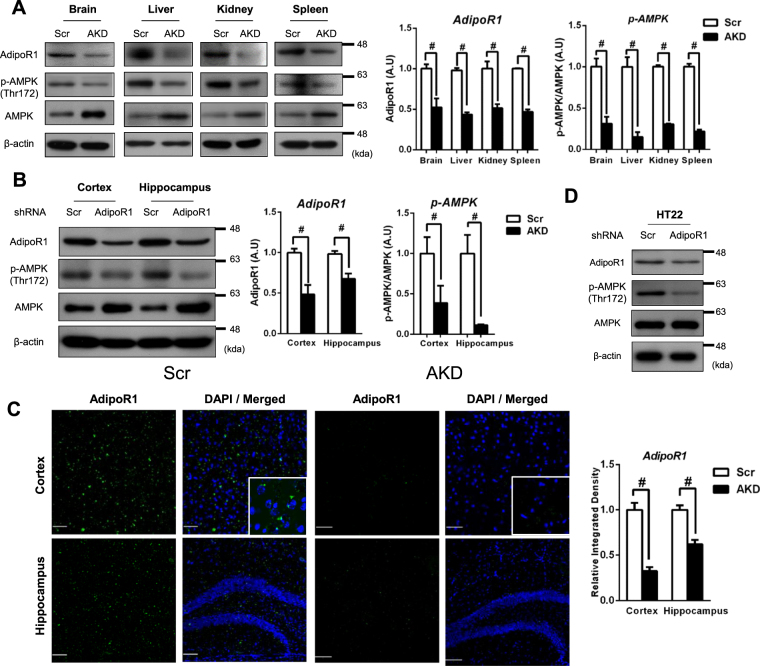
Confirmation of AdipoR1 expression in various organs. (**A**) AdipoR1, p-AMPK, and AMPK expression in the brain, liver, kidney and spleen. (**B**) Densitometry analysis of AdipoR1 and p-AMPK at 11 weeks after transfection with the PEI-shRNA complex. (**C**) Wide distribution of AdipoR1 in the cortex and hippocampus in Scr mice compared with AKD mice. (**D**) AdipoR1 expression in mouse hippocampal HT22 cells with or without AdipoR1 shRNA. Values indicate the mean ± SEM, n = 8–10, ^#^P < 0.001, Student’s t-test.

To assess the role of AdipoR1 in the CNS, we generated a polyethylenimine (PEI)-based gene therapy-mediated knockdown mouse model as previously reported^25^ and examined the phenotypes, such as the metabolic profile and spatial and learning memory (Fig. 1B). After 6-weeks of injections with the shRNA mixture, we examined the expression of AdipoR1 in various organs including the brain, liver, kidney and spleen. AdipoR1 expression was significantly decreased in 11-week-old AKD mice (Fig. 2A). AMP-activated protein kinase (AMPK), a master switch of peripheral metabolism, is directly regulated by AdipoR1^26^. AMPK is activated by upstream AMPK kinase (AMPKK), which phosphorylates threonine 172. Therefore, we investigated the expression of AMPK phosphorylation (thr172) in Scr and AKD mice. AMPK phosphorylation in AKD mice was significantly shifted to AMPK compared with that observed in the Scr mice (Fig. 2A). Specifically, the cortex and hippocampus were dissected from the whole brain, and the expression pattern of AdipoR1 in these regions was in line with that in the whole brain (Fig. 2B). In addition, we confirmed that the AdipoR1 expression in the cortex and hippocampus was clearly decreased in AKD mice (Fig. 2C). In the *in vitro* model, mouse hippocampal HT22 cells expressed AdipoR1, and both AdipoR1 and AMPK phosphorylation were decreased after transfection with AdipoR1 shRNA.

### AdipoR1 knockdown induces a metabolic dysfunction phenotype

**Figure 3:**
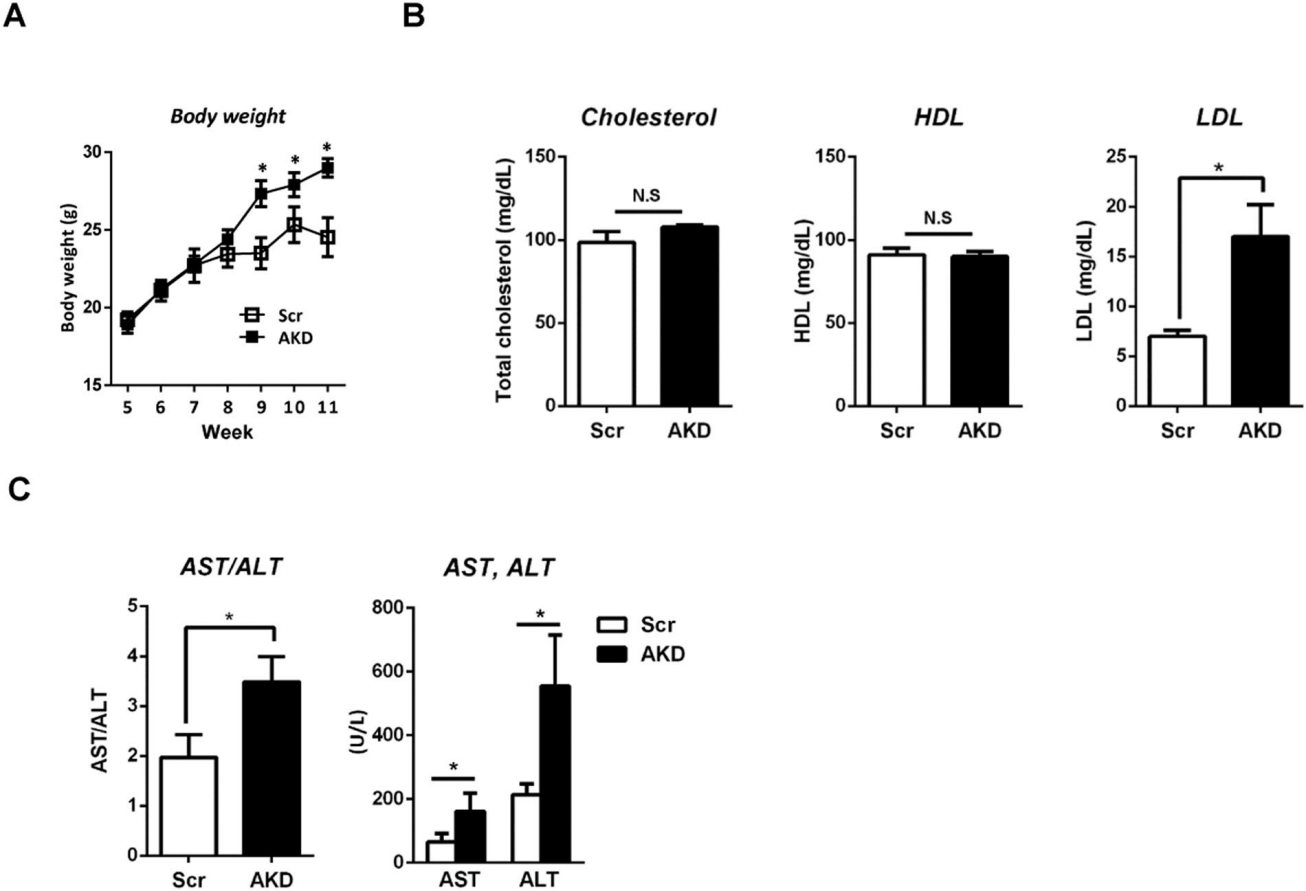
Metabolic profiling after PEI-shRNA plasmid complex transfection. (**A**) Body weight curves of scrambled control (Scr) and AdipoR1 knockdown (AKD) mice under a normal chow condition. (**B**) Plasma biochemistry in Scr and AKD mice. Mice were fasted overnight before the tissue and serum samples were obtained. The mice were 11 weeks old. Values indicate mean ± SEM, n = 8–10, *P < 0.01, Student’s t-test.

At week 9, AKD mice, compared with mice transfected with the Scr shRNA, showed a significant increase in body weight (Fig. 3A). During shRNA-mediated transfection, there were no differences in food intake between Scr and AKD mice (data not shown). At week 11, we euthanized the mice and analysed the plasma chemistry after overnight starvation. The total cholesterol and high-density lipoprotein (HDL) levels were similar between the Scr and AKD mice. However, levels of low-density lipoprotein (LDL), which may move lipids into artery walls and induce atherosclerosis, were significantly higher in AKD mice (Fig. 3B). In addition, AKD mice were exposed to hepatic dysfunction by elevation of the aspartate transaminase (AST) and alanine transaminase (ALT) levels and their ratio (Fig. 3C). Previous reports have shown that adiponectin receptor knockout (AdipoR1^−/−^) mice exhibit an abnormal body weight increase and plasma chemistry when administered a normal chow diet^23^. Our data showed that AdipoR1^−/−^ mice and AKD mice shared similar patho-phenotypes.

### AdipoR1 suppression induces spatial learning and memory impairment

**Figure 4:**
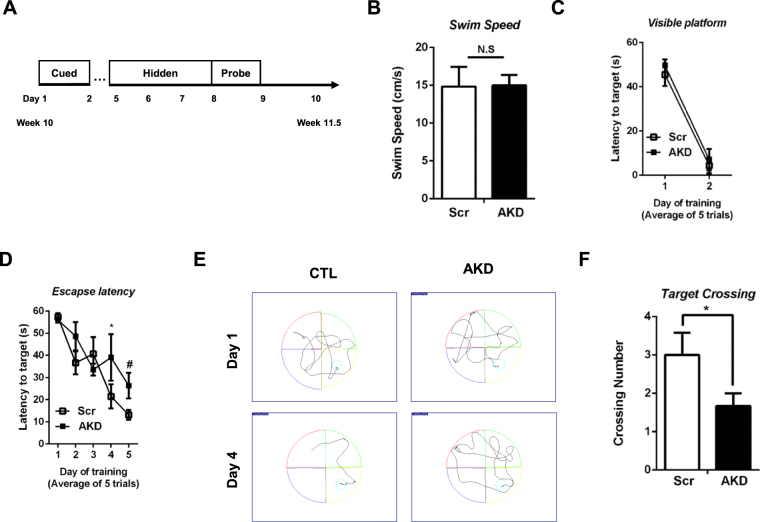
AdipoR1 silencing exacerbates memory impairments. (**A**) Schematic strategy to measure the memory impairment in AKD mice. (**B**) Scr and AKD mice showed no differences in locomotor activity. Both groups were exposed to the same testing conditions. Mean escape latency to reach the (**C**) visible platform and (**D**) hidden platform. (**E**) The trajectory map was recorded during the hidden platform test (Day 5). (**F**) AKD mice preferred the specific target quadrant where the hidden platform was located. Values indicate the mean ± SEM, n = 8–10, ^#^P < 0.001, Student’s t-test.

AMPK activation, which is directly regulated by AdipoR1, has been reported to attenuate spatial memory impairment^27–29^. Because AdipoR1 is upstream of AMPK on the axis, we hypothesized that AKD mice are prone to memory impairment. To test this hypothesis, we used the Morris water maze test as depicted in Fig. 4A. First, the visual field and locomotor activity were tested to rule out effects of potential physical handicaps. We confirmed that both Scr and AKD mice had a normal visual field and locomotor activity during the training session. (Fig. 4B,C). Next, the mice were exposed to opaque water to hide the platform. After 4 days of training, AKD mice exhibited difficulties finding the platform (Fig. 4D and E). To examine memory function, probe trials were performed by removing the platform on day 8. In the probe trials, the frequency of crossing the previous location of the platform was significantly lower in AKD mice than in the Scr mice (Fig. 4F). Hence, our data demonstrated that decreased expression of AdipoR1 worsened spatial and learning memory.

### AdipoR1 down-regulation aggravates neuronal apoptosis

**Figure 5:**
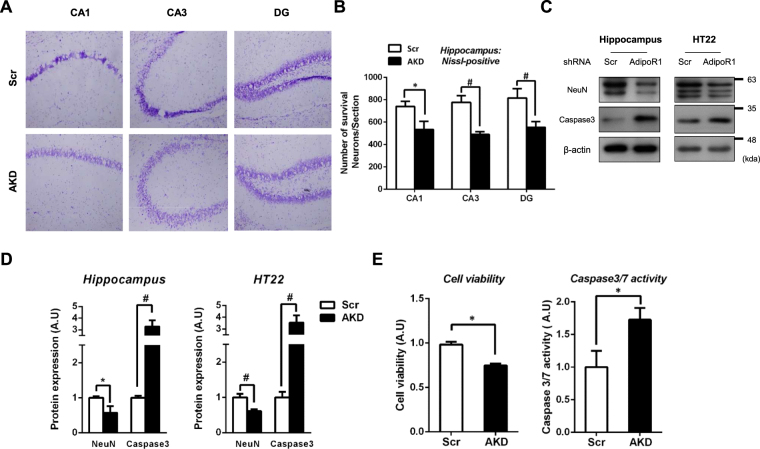
Neurodegenerative phenotypes in AKD mice. (**A**) Cresyl violet staining to (**B**) quantify the Nissl-positive cells and to detect the morphology in the Scr and AKD mouse hippocampal CA1, CA3 and DG regions. (**C**) Western blot analysis of neuronal nuclei (NeuN) and caspase 3 (Caspase 3) in Scr and AKD mice. (**D**) Densitometry analysis of NeuN and Caspase 3 (**E**) Cell viability and caspase 3/7 activity after 3 days of Scr and AdipoR1 shRNA transfection. Values indicate the mean ± SEM, n = 8–10, *P < 0.01, ^#^P < 0.001, Student’s t-test.

Examining neuronal cell death-dependent spatial learning and memory impairment is one way to approach the investigation of neurodegenerative diseases. To verify that the spatial learning and memory impairment were hippocampal-dependent, we stained the hippocampus with cresyl violet, which stains Nissl bodies purple. As expected, the survival of neurons in AKD hippocampal regions, including the cornu ammonis (CA) 1, CA3 and the dentate gyrus (DG), was markedly decreased (Fig. 5A,B). Next, we determined the expression of the neuronal marker neuronal nuclei (NeuN) and apoptotic initiator caspase 3 in the hippocampus and mouse hippocampal HT22 cells. Compared with Scr mice, AKD mice exhibited decreased NeuN and increased caspase 3 expression (Fig. 5C,D). To further demonstrate the effects of AdipoR1 suppression, we used the cell viability and caspase 3/7 activity assay to verify apoptosis induced by AdipoR1 suppression in vitro. A single transfection of AdipoR1 shRNA dramatically decreased cell viability and increased caspase 3/7 activity. Unexpectedly, ADPN knockout mice have been reported to exhibit neuronal apoptosis and spatial learning and memory impairment, as compared with that in WT mice^22^. Therefore, our data suggested that the ADPN-AdipoR1 axis plays an important role in spatial learning and memory as well as neuronal apoptosis.

### AdipoR1 suppression exacerbates Alzheimer’s disease-like pathologies

**Figure 6:**
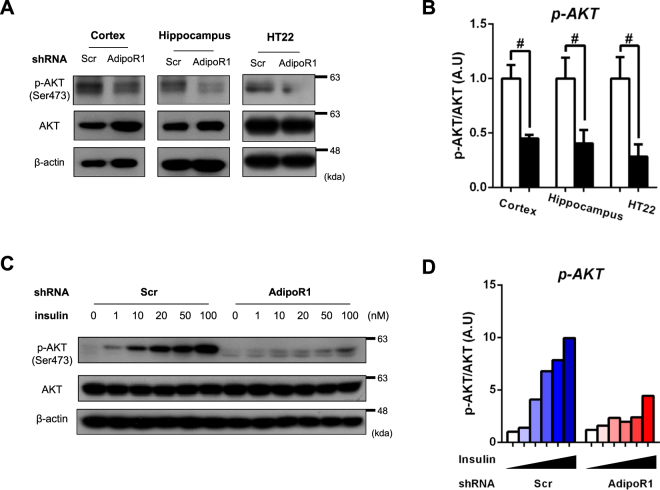
Insulin signalling dysfunction in AKD mice. (**A**) p-AKT and AKT expression in the mouse cortex, hippocampus and mouse hippocampal HT22. (**B**) Densitometry analysis of p-AKT and AKT at 11 weeks after transfection with the PEI-shRNA complex and after 3days of Scr and AdipoR1 shRNA transfection. (**C**) Western blot analysis of p-AKT and AKT to check insulin sensitivity by dose-dependent manner for 10 min in Scr and AdipoR1 shRNA transfection. (**D**) Densitometry analysis of p-AKT and AKT. Values indicate the mean ± SEM, n = 3–5, *P < 0.01, ^#^P < 0.001, Student’s t-test.

Present study showed that AKD mice have exhibited similar phenotypes including spatial learning and memory impairment and neuronal apoptosis compared with ADPN^−/−^ mice^22^. Next, we approached to pathogenic mechanisms underlying the cognitive impairment of spatial learning and memory as well as neurodegeneration. Previous studies that ADPN-AdipoR1 signalling pathway have a major role of insulin-sensitizing effect via activating protein kinase B (PKB or AKT)^18^. As expected, basal level of AKT phosphorylation was down regulated in mouse cortex, hippocampus and mouse hippocampal HT22 cells (Fig. 6A,B). To investigate whether AdipoR1 deficiency affects insulin-responsive phenotype, we treated insulin in HT22 cells by dose-dependent manner. Interestingly, AKD group exhibited reduced AKT phosphorylation compared with Scr group (Fig. 6C,D). Furthermore, we hypothesized that ADPN^−/−^ mice and AKD mice will share patho-phenotype; AD-like neuropathologies such as proteinopathies and neuroinflammation on the basis of previous reports^22^. Preferentially, we checked abnormal protein accumulation such as Aβ production and tau hyperphosphorylation. Aβ production was dramatically increased in AKD mice (Supplementary Fig. 1a). Next, we investigated whether glycogen synthase kinase 3β (GSK3β) form is active or not due to its role for tau hyperphosphorylation^30^. Although expression level of GSK3β are highly up regulated in both regions of AKD mice, phosphorylation of GSK3β at serine 9, considering an inhibitory form, was decreased in the cortex of AKD mice except for hippocampus (Supplementary Fig. 1a). Sequentially, tau protein phosphorylation at serine 396/404 (paired helical filaments or PHF-1) in was highly regulated in both regions of AKD mice, total tau (Tau5) level also increased in AKD mice (Supplementary Fig. 1a). Next, we confirmed neuroinflammatory phenotypes using microglial marker and pro-inflammatory cytokines. Ionized calcium-binding adapter molecule 1 (Iba-1) and tumor-necrosis factor alpha (TNF-α) level were up-regulated in AKD mice compared with Scr mice (Supplementary Fig. 1b). This data is consistent with a recent finding that AdipoR1 knockdown in microglial cell line BV-2 exhibited M1 polarization, pro-inflammatory phenotype, under the basal condition^31^. In summary, AdipoR1 knockdown have shown neurodegeneration-mediated memory dysfunction as well as global AD-like pathologies including insulin signalling dysfunction, proteinopathies and neuroinflammation.

## Discussion

Neurodegenerative diseases have been regarded as a type 3 diabetes (T3D). The CNS pathology shares phenotypic aspects with type 2 diabetes (T2D), such as insulin resistance and glucose intolerance^32,33^. Patients with T2D experience mild cognitive impairment; therefore, peripheral metabolic dysfunction is considered a risk factor for neurodegenerative disease^34,35^. In fact, the canonical pathologies of neurodegeneration, such as protein aggregation, inflammation, and increase in reactive oxygen species (ROS), decrease aspects of energy metabolism such as insulin signalling and glucose metabolism in the brain^36–39^. According to this approach, insulin-sensitizing agents, such as thiazolidinediones (TZDs) improve AD pathogenesis^40–42^. Therefore, ameliorating energy metabolism has been considered as a therapeutic target to prevent AD pathogenesis^43,44^.

Here, we used polyethylenimine (PEI)-based shRNA transfection to knock down AdipoR1 and to confirm the acute effect of AdipoR1 in mice. In a previous report, *Bjursell*, *M*. *et al*. have found differences in body weight and plasma chemistry between WT and AdipoR1^−/−^ mice starting at 15 weeks under feeding of normal chow^23^. In AKD mice, 3 weeks of shRNA injection treatments induced an increase in body weight, as compared with that of the Scr mice. To confirm the similar patho-phenotypes observed in AdipoR1^−/−^ mice, we evaluated the plasma chemistry in both Scr and AKD mice. First, we determined whether PEI itself affected the metabolic profile, including the body weight and plasma biochemistry; however, there were no significant differences between the PEI only and Scr group (data not shown). To examine the metabolic dysfunction, we examined dyslipidaemia and hepatic dysfunction on the basis of plasma chemistry. There were no significant differences in the total cholesterol or high-density lipoprotein (HDL); however, the low-density lipoprotein (LDL) levels were significantly higher in the AKD mice. Aspartate transaminase (AST) and alanine transaminase (ALT) and their ratio were examined as indicators of hepatic dysfunction and were dramatically elevated in the AdipoR1-suppression group. Thus, we confirmed that the phenotypes of AKD mice are in line with those of the AdipoR1^−/−^ mice.

Recently, the role of ADPN in neuropathology have been illuminated. Ng, R. C. *et al*. have shown global neuropathologies, as well as spatial memory impairment in adiponectin knockout (ADPN^−/−^) mice. In fact, 18-month-ADPN^−/−^ mice exacerbated proteinopathies such as Aβ production and hyperphosphorylated tau, neuroinflammation, neuronal cell loss and insulin resistance along with impairment in spatial learning and memory^22^. Zhang *et al*. have shown that adiponectin deficiency exhibited reduced total dendritic length and number of branches in both early and late-born neurons^45^. In addition, immature dendritic spine density was highly expressed in ADPN^−/−^ mice. From ADPN-AdipoR1 axis perspective, we hypothesized that loss of AdipoR1 would result in spatial learning and memory impairments as well as neurodegeneration. We then conducted the Morris water maze test to verify whether AdipoR1 suppression leads to hippocampus-dependent memory dysfunction. Our data suggested that memory dysfunction was impaired by AdipoR1 shRNA, and the impairment was dependent on the hippocampus- and medial entorhinal cortex (MEC). In addition, a neuronal cell death is considered as a major phenotype of neurodegenerative disease, including the loss of particular subsets of neurons^46^. Therefore, we examined the hippocampal neuronal density. Hippocampal neuronal loss was significantly higher in AKD mice. To further confirm this neuronal loss, we examined the expression of NeuN, a neuronal marker, and caspase 3, a final executor of apoptosis. Both hippocampus and mouse hippocampal HT22 cells exhibited a decrease in NeuN expression and an increase in caspase 3. Kondo, K. *et al*. have found that ADPN relieved ischaemia and reperfusion (I/R)-induced caspase 3 activation^47^. Lee, E. B. *et al*. have suggested that people with ADPN deficiency may be vulnerable to kainic acid exposure^21^. Previous studies have supported our findings that the ADPN-AdipoR1-AMPK axis is a major neuroprotective pathway as well as a major pathway involved in metabolic syndromes.

As we mentioned, Ng, R. C. *et al*. have shown that ADPN^−/−^ mice displayed AD-like pathologies such as abnormal protein aggregation, brain inflammation and insulin signalling dysfunction^22^. On the basis of the previous findings, we asked whether ADPN^−/−^ mice and AKD mice shares pathological phenotypes. Fundamentally, ADPN signalling pathway had been identified as a hormone which has a role of insulin-sensitizing effect by activating AKT phosphorylation at serine 473^48,49^. To verify that AdipoR1 deficiency attenuates insulin signalling pathway, we first checked basal level of AKT phosphorylation at serine 473. Mouse cortex, hippocampus and hippocampal HT22 cell have shown reduced AKT phosphorylation in AKD group. Additionally, we confirmed that insulin sensitivity was decreased in HT22 cells after treating insulin by dose-dependent manner. Furthermore, other pathological status of neurodegeneration-mediated memory impairment such as Aβ oligomer, tau hyperphosphorylation and neuroinflammation was highly up regulated in AKD group, suggesting that ADPN-AdipoR1 axis has a central role of preventing AD-like pathologies.

The expression pattern of AdipoR1 is dependent on pathological status. In fact, obesity-linked insulin resistance and diabetes down-regulate AdipoR1 expression^18^. Yamauchi, T. *et al*. have reported that adenovirus-mediated AdipoR1 overexpression rescues insulin resistance and diabetes^50^. Additionally, in an AD mouse model, APP/PS1, AdipoR1 protein expression are significantly down-regulated^51^; thus, facilitating the expression of AdipoR1 is a logical way to regulate peripheral or central pathological models. We have previously reported that osmotin, which is homologous to ADPN, exhibits a neuroprotective role against ethanol-induced apoptosis in the developing rat brain, via AdipoR1^52^. Similarly, the importance of AdipoR1 has also been highlighted in a study emphasizing the role of osmotin in improving AD pathology by inhibition of SREBP2^53^. Because we found a fundamental role for AdipoR1 in the CNS, our data strongly support ADPN- or ADPN-like ligand (osmotin)-mediated neuroprotection.

In summary, we found that decreased AdipoR1 expression causes spatial learning and memory impairment and AD-like pathologies, thus suggesting that the ADPN- or ADPN-like ligand-AdipoR1 axis should be considered as a treatment target for AD.

## Materials and Methods

### Chemicals

Anti-adiponectin receptor 1 (Abcam), anti–p-AMPK, anti-AKT (Cell Signaling), anti-β-actin, anti-AMPK, anti-caspase 3, anti-p-AKT, anti-Tau, anti-p-tau, anti-Aβ, anti-GFAP, anti-TNF-α, FITC-labelled goat-anti rabbit (Santa Cruz), anti-NeuN (Millipore), anti-iba-1 (Wako), insulin (Sigma), branched-PEI (Sigma), D-(+)- glucose solution (Sigma), DPX (Sigma), AdipoR1 shRNA plasmid (Qiagen), phosphatase inhibitor, protease inhibitor cocktail (GenDEPOT), a protein assay kit (Bio-Rad), skim milk (BD Difco), O.C.T. compound (Sakura), ECL solution (ATTO), and fluorescence mounting medium (Agilent Technologies) were purchased from the indicated manufacturers.

### Establishing the PEI-based shRNA-mediated AdipoR1 knockdown strategy

To establish the PEI-based shRNA-mediated knockdown, we optimized the composition and condition of the PEI-shRNA mixture on the basis of a previous report. PEI and the shRNA plasmid were dissolved in 10% glucose for 5 min at room temperature. Next, the mixtures were transferred to one tube and incubated for 15 min (Fig. 1A). Finally, the PEI-shRNA mixture was introduced into mice through intravenous injection once weekly, from week 5 to week 11 (Fig. 1B).

### Mouse strain

Male wild-type C57BL/6J mice (16–18 g, 5 weeks old) were purchased from Jackson Laboratory (Bar Harbor, ME, U.S.A). The mice were acclimated for 1 week in the university animal housing under a 12-h/12-h light/dark cycle at 23 °C with 60 ± 10% humidity and were provided with food and water ad libitum. The maintenance and treatment of the mice were carried out in accordance with the animal ethics committee (IACUC) guidelines issued by the Division of Applied Life Sciences, Department of Biology at Gyeongsang National University, South Korea. All efforts were made to minimize the number of mice used and their suffering. The experimental methods with mice were carried out in accordance with the approved guidelines (Approval ID: 125), and all experimental protocol were approved by the animal ethics committee (IACUC) of the Division of Applied Life Sciences, Department of Biology at Gyeongsang National University, South Korea.

### Morris water maze test

The Morris water maze test was conducted on the basis of previously reported methods, with some modifications^53,54^. On the day of training, each mouse (n = 8–10 per group) was allowed to freely float in the tank and to find the visible platform. Four training trials were conducted within 60 s per day for 2 days. Mice that took longer than 60 s to find the platform were manually moved to the platform and were left there for 5 s. The hidden platform test was conducted with the platform located in the opposite quadrant in opaque water for 5 days after a resting phase of 2 days. Probe trials used the hidden platform test to assess memory retention by allowing the mice to freely float in the opaque water for 60 s. From this test, data, including the time to reach the visible platform, swim speed, escape latency and target crossing frequency, were analysed and recorded.

### Western blot analysis

Animals were sacrificed after 6 weeks of PEI-shRNA plasmid complex injection. Total protein from the organs was extracted using RIPA buffer (50 mM sodium chloride, 1% Triton X-100, 1% sodium deoxycholate, 0.1% SDS, 50 mM Tris-HCl, pH 7.5, and 2 mM EDTA) containing phosphatase and protease inhibitor cocktail. The protein concentration was quantified with a Bradford assay. Equal amounts of protein (25 µg) were resolved by SDS-PAGE, transferred to a PVDF membrane, and blocked in 5% (w/v) skim milk before incubation with primary antibodies overnight at 4 °C at 1:1,000–1:10,000 dilutions. The proteins were detected using an ECL detection reagent according to the manufacturer’s instructions. The X-ray films were scanned, and the optical densities of the bands were analysed via densitometry using the computer-based ImageJ program.

### Immunofluorescence assays

Mice were anaesthetized and transcardially perfused with 1X ice-cold PBS and 4% paraformaldehyde (PFA). After 48 h of fixation, the brains were transferred to 20% sucrose in 1X PBS for 24 h. Brains were implanted in matrix containing O.C.T compound and frozen in liquid nitrogen. The brain slices were coronally sectioned at a 20-μm thickness with a CM 1950 cryostat (Leica). Tissue-containing slides were washed twice for 15 min in 1X PBS, and proteinase K solution was then added for 5 min at 37 °C to retrieve the antigen. Subsequently, the slides were incubated for 90 min in blocking solution containing normal goat serum and 0.1% Triton X-100 in PBS. Primary antibodies were applied at 4 °C overnight. The next day, the slides were washed twice with 1X PBS for 10 min and incubated with a goat-anti rabbit antibody labelled with FITC for 90 min. After the slides were washed in PBS, they were treated with 4′,6′ -diamidino-2-phenylindole (DAPI) for 10 min and mounted with glass coverslips using mounting medium. AdipoR1 was observed with a confocal laser-scanning microscope (Fluoview FV 1000, Olympus). To measure the relative integrated density, the signal and area were obtained by using the ImageJ analysis program.

### Nissl staining

Nissl bodies were stained with cresyl violet. Briefly, brain slices were washed with tap water, immersed in 100% ethanol, and incubated in 100% xylene for 15 min and 100% ethanol for 10 min. Next, the slices were incubated with 0.1% cresyl violet for 15 min, washed in 70% ethanol, immersed with differentiation solution (2 drops of glacial acetic acid in 95% ethanol), cleared in xylene for 3 min and mounted with DPX mounting medium under a coverslip. Nissl-positive cells in the hippocampus were measured with the ImageJ analysis program.

### Cell cultures, shRNA transfection, cell viability and caspase 3/7 activity assays

Mouse-derived hippocampal HT22 cells were grown in Dulbecco’s modified Eagle’s medium (DMEM) containing 10% FBS and 1% antibiotic-antimycotic in a humidified 5% (v/v) CO_2_ incubator at 37 °C. Cells were seeded on the plate at a density of 1.5 × 10^5^ cells/ml, and AdipoR1 shRNA (Qiagen) was transfected using Lipofectamine 3000 according to the manufacturer’s instructions. AdipoR1 knockdown was measured after 72 h of transfection by western blotting. After 72 h of transfection, physiological changes such as cell viability and apoptosis were measured with 3-(4,5-dimethylthiazol-2-yl)-2,5-diphenyltetrazolium (MTT) assays and Caspase-Glo^TM^ 3/7 assays (Promega) according to the manufacturer’s instructions.

### Statistical analysis

For western blots, the optical densities are expressed as the mean + s.e.m. with arbitrary units. Prism 6 (GraphPad Software, San Diego, CA, USA) was used to perform Student’s t-tests on data from the western blots and morphological analyses. The data were considered significantly significant at P < 0.05 and 0.001.

## Electronic supplementary material

Supplementary data
