# Supplementary material for: Suppression of adiponectin receptor 1 promotes memory dysfunction and Alzheimer’s disease-like pathologies
Source: Sci Rep. 2017 Sep 29;7:12435. doi: 10.1038/s41598-017-12632-9 (PMC5622055; doi:10.1038/s41598-017-12632-9)

# Suppression of adiponectin receptor 1 promotes memory dysfunction and Alzheimer's disease-like pathologies

Min Woo Kim[1,∮], Noman bin Abid [1, ∮], Myeong Hoon Jo[1], Min Gi Jo[1], Gwang Ho Yoon[1], and Myeong Ok Kim[1, *]

[1] Division of Life Science and Applied Life Science (BK 21 plus), College of Natural Sciences, Gyeongsang National University, Jinju, Republic of Korea

[∮] These authors contributed equally to this work.

*** Correspondence:**
Prof. Myeong Ok Kim, Ph.D.

Head of Neuroscience Pioneer Research Center,

Head of Brain-Metabolic Neurodegenerative Disease Center,

Division of Life Science and Applied Life Science, College of Natural Sciences,

Gyeongsang National University, Jinju, 660-701, South Korea

Tel.: +82-55-772-1345

Fax: +82-55-772-1349

To whom correspondence should be addressed: E-mail: mokim@gnu.ac.kr

Supplementary fig. 1

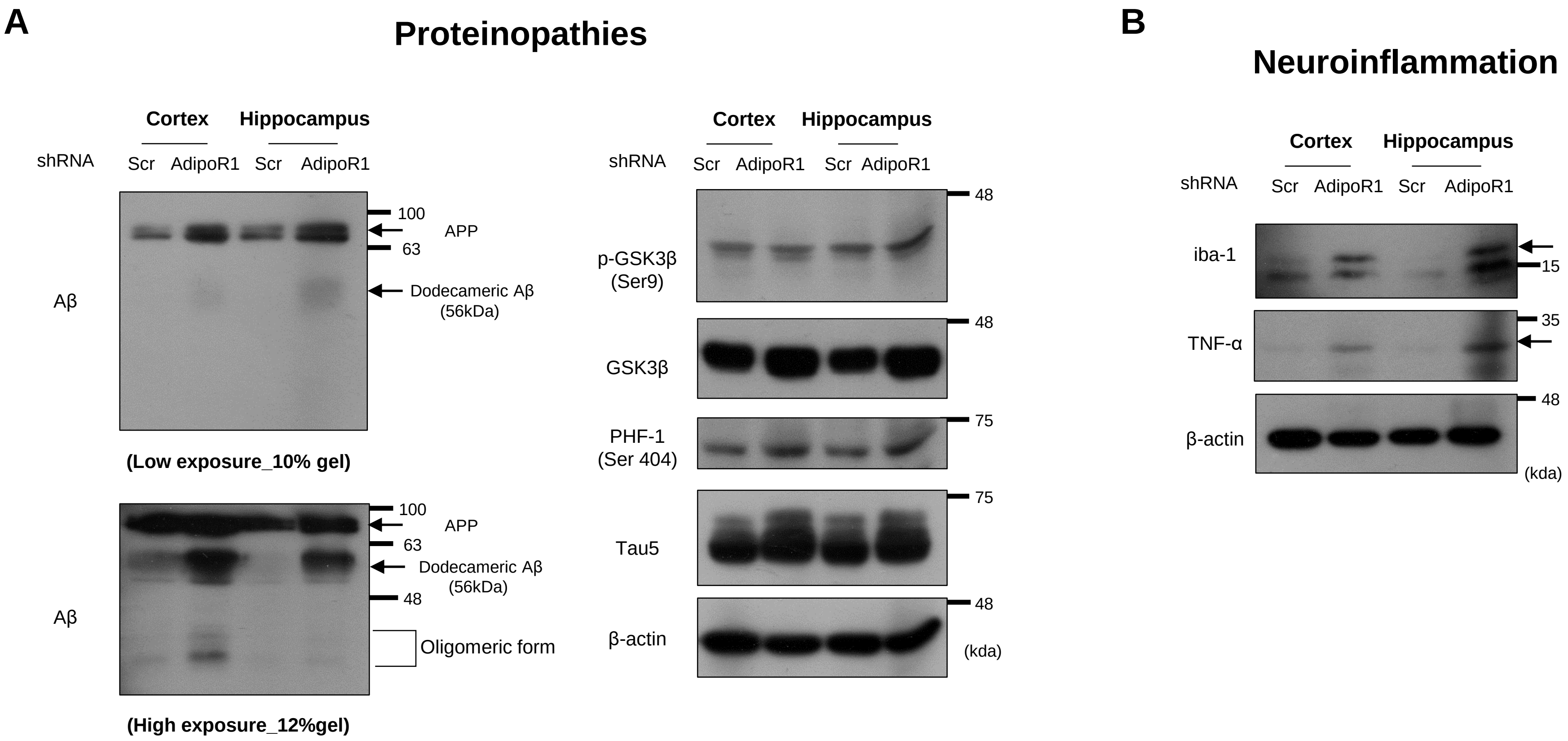


**Supplementary figure. 1** AD-like pathologies in AKD mice (A). Oligomeric Aβ, GSK3β and hyperphosphorylated tau expression in the AKD mice. (B) Neuroinflammatory marker including iba-1 and TNF-α in the AKD mice

**Complete Immunoblots:**

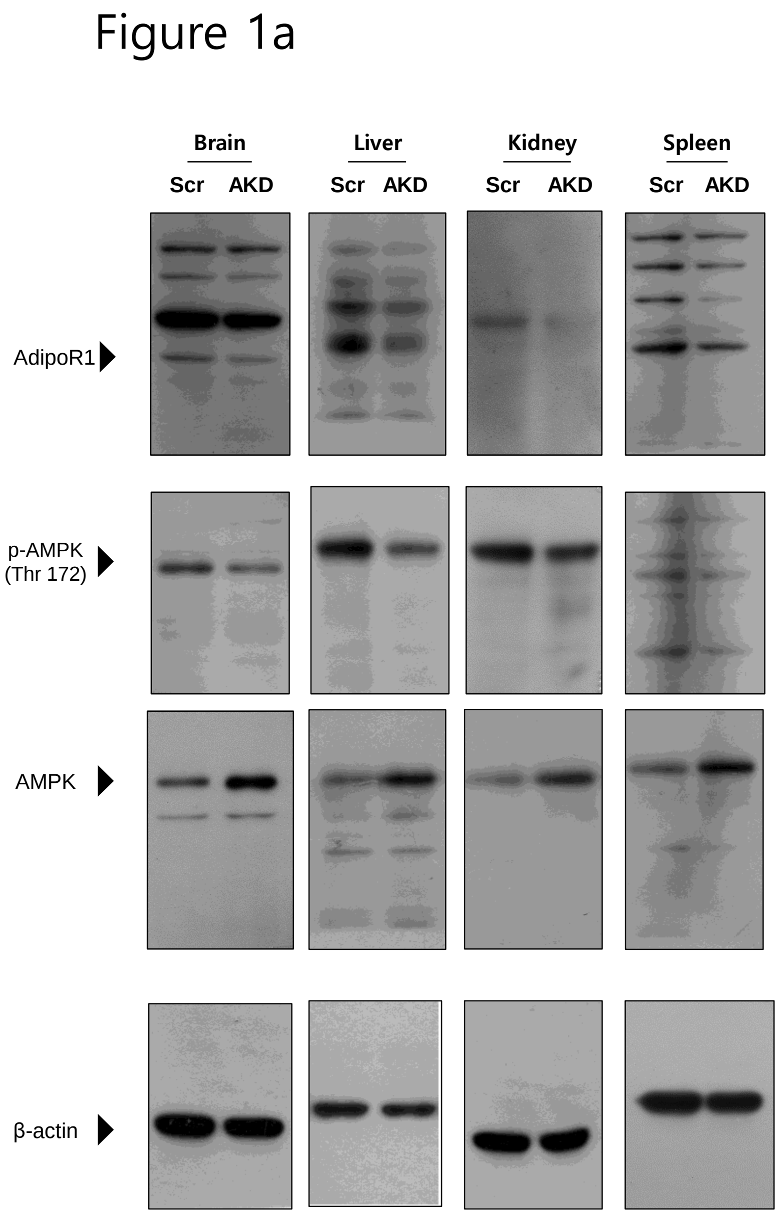


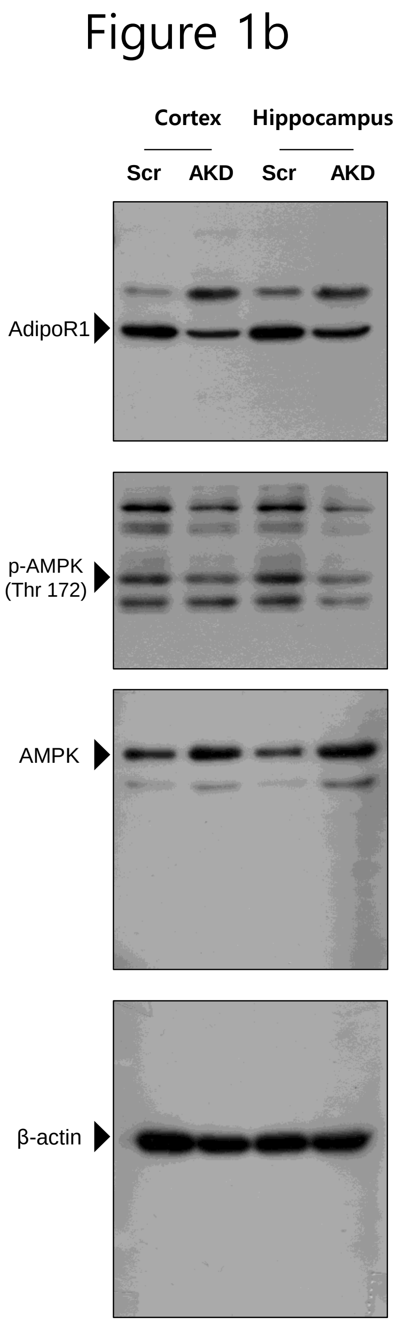


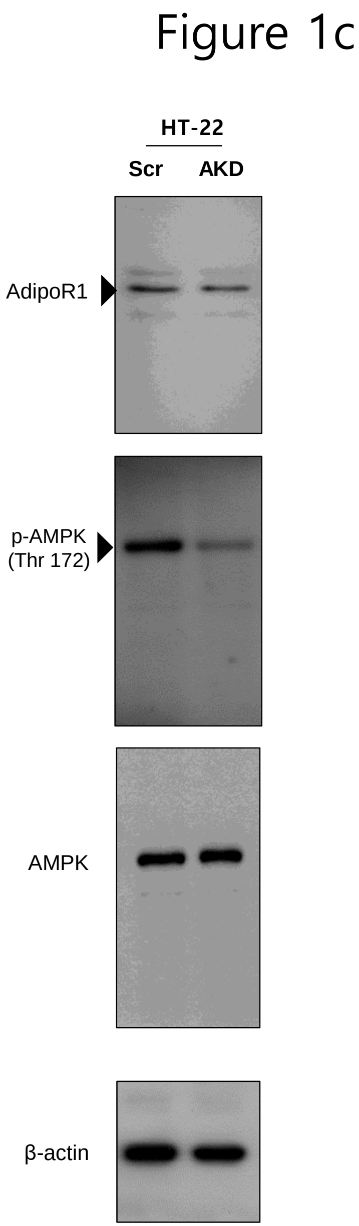

Figure 5c

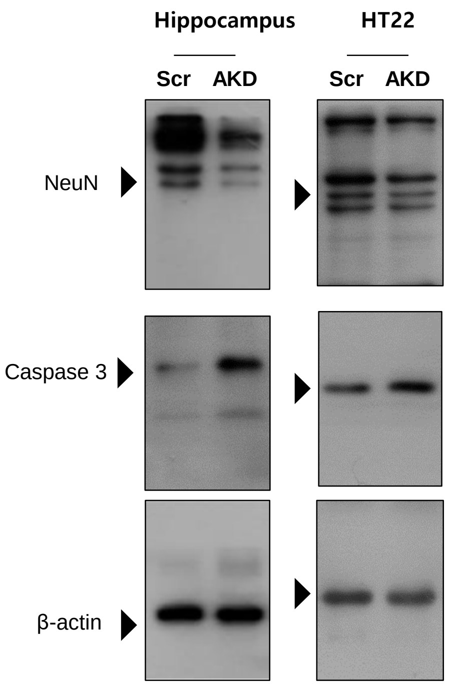

Figure 6a

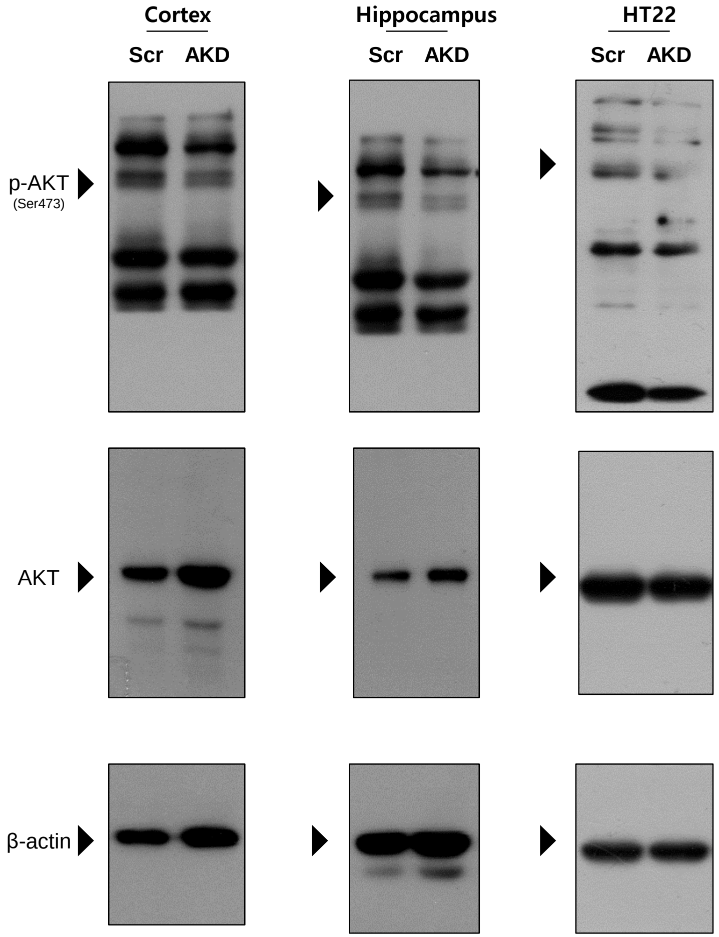


Figure 6c

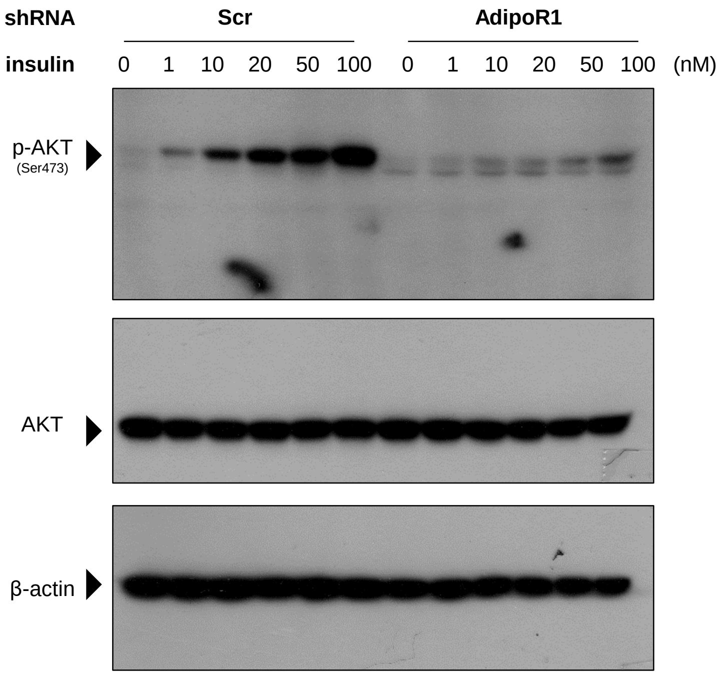

## Supplementary fig. 1a

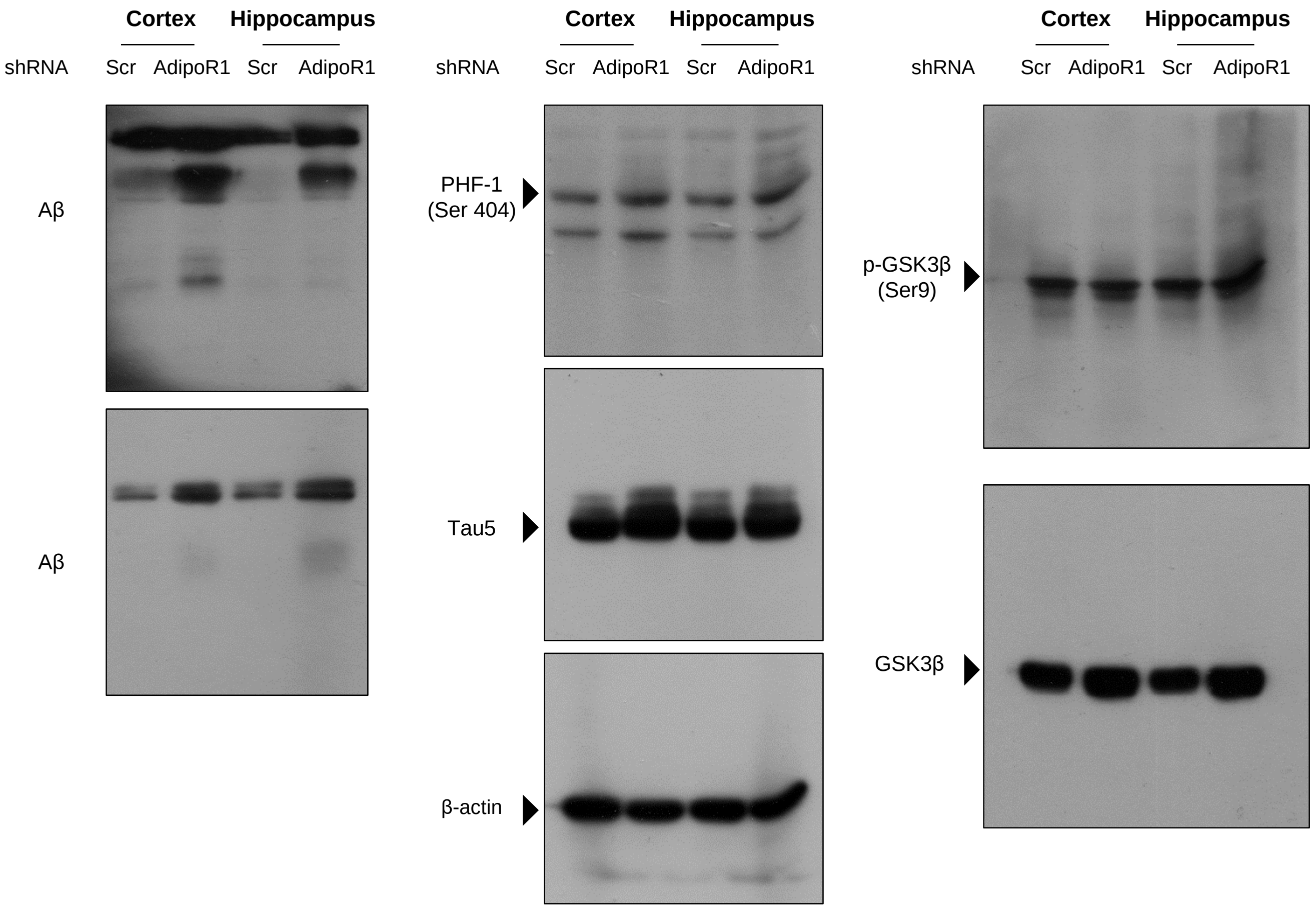


## Supplementary fig. 1b

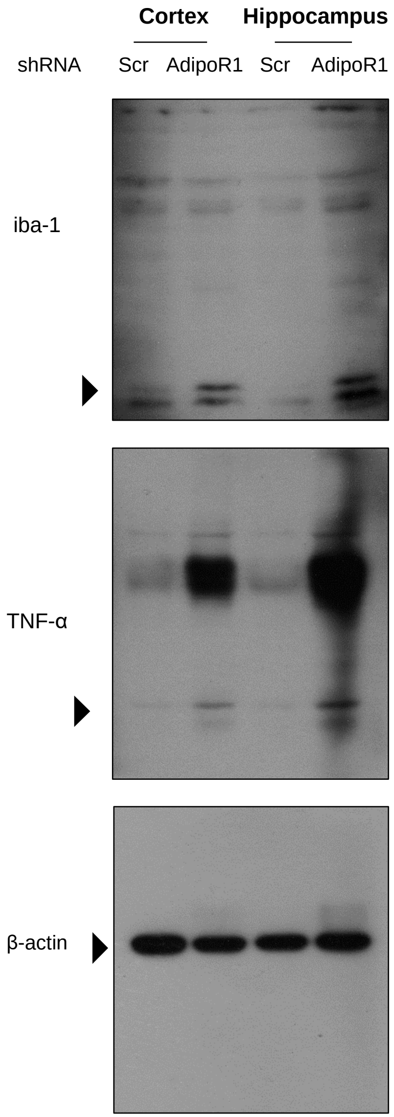

Supplement: Supplementary file 1 — Supplementary data [file 41598_2017_12632_MOESM1_ESM.pdf]
